# Induction of Identical IgG HIV-1 Envelope Epitope Recognition Patterns After Initial HIVIS-DNA/MVA-CMDR Immunization and a Late MVA-CMDR Boost

**DOI:** 10.3389/fimmu.2020.00719

**Published:** 2020-04-28

**Authors:** Agricola Joachim, Mohamed I. M. Ahmed, Georgios Pollakis, Lisa Rogers, Verena S. Hoffmann, Patricia Munseri, Said Aboud, Eligius F. Lyamuya, Muhammad Bakari, Merlin L. Robb, Britta Wahren, Eric Sandstrom, Charlotta Nilsson, Gunnel Biberfeld, Christof Geldmacher, Kathrin Held

**Affiliations:** ^1^Department of Microbiology and Immunology, Muhimbili University of Health and Allied Sciences (MUHAS), Dar es Salaam, Tanzania; ^2^Division of Infectious Diseases and Tropical Medicine, University Hospital, LMU Munich, Munich, Germany; ^3^German Centre for Infection Research (DZIF), Partner Site Munich, Munich, Germany; ^4^Faculty of Health and Life Science, Institute of Infection and Global Health, University of Liverpool, Liverpool, United Kingdom; ^5^NIHR Health Protection Research Unit in Emerging and Zoonotic Infections (HPRU EZI), Liverpool, United Kingdom; ^6^Institute for Medical Information Processing, Biometry, and Epidemiology, LMU Munich, Munich, Germany; ^7^Department of Internal Medicine, Muhimbili University of Health and Allied Sciences (MUHAS), Dar es Salaam, Tanzania; ^8^Tanzania Ministry of Health, Community Development, Gender, Elderly, and Children, Dodoma, Tanzania; ^9^Walter Reed Army Institute of Research (WRAIR), Rockville, MD, United States; ^10^Henry M. Jackson Foundation for the Advancement of Military Medicine, Bethesda, MD, United States; ^11^Department of Microbiology, Tumor and Cell Biology, Karolinska Institutet, Stockholm, Sweden; ^12^Department of Clinical Science and Education, Karolinska Institutet, Sodersjukhuset, Stockholm, Sweden; ^13^Department of Laboratory Medicine, Karolinska Institutet, Huddinge, Sweden; ^14^The Public Health Agency of Sweden, Solna, Sweden; ^15^Department of Global Public Health, Karolinska Institutet, Stockholm, Sweden

**Keywords:** human immunodeficiency virus 1 (HIV-1), vaccine, envelope (Env), envelope-specific antibody response, epitope variants, immunogen structure, immunogen sequence, linear peptide array

## Abstract

In the RV144 trial, to date the only HIV-1 vaccine efficacy trial demonstrating a modestly reduced risk of HIV-1 acquisition, antibody responses toward the HIV Envelope protein (Env) variable (V) 2 and V3 regions were shown to be correlated with a reduced risk of infection. These potentially protective antibody responses, in parallel with the vaccine efficacy, however, waned quickly. Dissecting vaccine-induced IgG recognition of antigenic regions and their variants within the HIV-1 Env from different vaccine trials will aid in designing future HIV-1 immunogens and vaccination schedules. We, therefore, analyzed the IgG response toward linear HIV-1 Env epitopes elicited by a multi-clade, multigene HIVIS-DNA priming, and heterologous recombinant modified vaccinia virus Ankara (MVA-CMDR) boosting regimen (HIVIS03) and assessed whether a late MVA-CMDR boost 3 years after completion of the initial vaccination schedule (HIVIS06) restored antibody responses toward these epitopes. Here we report that vaccination schedule in the HIVIS03 trial elicited IgG responses against linear epitopes within the V2 and V3 tip as well as against the gp41 immunodominant region in a high proportion of vaccinees. Antibodies against the V2 and gp41 Env regions were restricted to variants with close homology to the MVA-CMDR immunogen sequence, while V3 responses were more cross-reactive. Boosting with a late third MVA-CMDR after 3 years effectively restored waned IgG responses to linear Env epitopes and induced targeting of identical antigenic regions and variants comparable to the previous combined HIVIS-DNA/MVA-CMDR regimen. Our findings support the notion that anti-HIV-1 Env responses, associated with a reduced risk of infection in RV144, could be maintained by regular boosting with a single dose of MVA-CMDR.

## Introduction

With an estimated 1.7 million new HIV infections worldwide in 2018 as reported by the WHO, HIV-1 remains a global health challenge. To stop the on-going HIV epidemic, a safe and effective HIV vaccine is urgently required. So far, the virus' immune evasion mechanisms have hampered these attempts. One of the main challenges lies in the extraordinarily high mutation rate of HIV, which results in high antigenic variability of the HIV-1 Envelope (Env) protein, the only viral antigen exposed on the surface of the viral particle. The HIV-1 Env comprises three gp120-gp41 heterodimers, together forming a meta-stable trimer, which is well-shielded from the immune system by N-linked glycans (1, 2).

Even though 10–50% of chronically HIV-1 infected individuals, both adults and children, develop broadly neutralizing antibodies (bnAbs) against the Env (3–5), and 1–2% of naturally infected individuals are so-called elite neutralisers with very high cross-clade activity (6), current immunization regimens have not succeeded in inducing such broad and potent HIV-1-neutralizing antibodies (7). Non-neutralizing antibodies binding to the HIV-1 Env, however, might also have the potential to protect against HIV-1 infection, as demonstrated by the analysis of immune correlates of infection risk of the RV144 HIV-1 vaccine efficacy trial, whereby modest protection of 31% was shown to correlate with specific binding antibody responses to the HIV Env (8–10). IgG antibodies to the HIV-1 Env variable (V) regions V1 and V2, as well as V3, were found to correlate with a reduced risk of HIV-1 infection, while the presence of IgA Env-binding antibodies was associated with an increased risk of infection (9, 10). Antibody responses to other linear epitopes of the HIV-1 Env gp120 did not correlate with infection risk (10), which might be due to the fact that many of these regions are not accessible on a native HIV-1 Env trimer (11). Viral sieve analyses showed that the RV144 vaccine regimen induced selection of viral variants with point mutations in the V2 and V3 regions, indicating that strain-specific V2 and V3 antibodies drove viral mutation to escape the vaccine-induced immune response against HIV-1 (12, 13). In addition, for a rhesus monkey adenovirus/poxvirus vaccine model, vaccine protection against simian immunodeficiency virus (SIV) challenges correlated with the presence of Env V2-specific binding antibodies (14). Vaccine efficacy of RV144, however, declined over time, with a cumulative vaccine efficacy of 60% at 6 months and 29% at 42 months after the final vaccination (15). The parallel waning of RV144-induced antibody responses toward the HIV-1 envelope, including anti-V2 responses (16, 17), suggests a link between declining anti-Env antibodies and declining vaccine efficacy. The exact mechanism by which these vaccine-induced antibodies might reduce the risk of HIV-1 infection is unclear; yet, monoclonal antibodies from RV144 vaccinees targeting the V2 region have been shown to bind HIV-1 infected cells and to mediate antibody-dependent cellular cytotoxicity (ADCC) activity *in vitro* (18).

The V3 region, part of the chemokine receptor binding region, is the least variable of the Env V regions, as the amino acid sequence variability is restricted to the crown of the V3 loop and length and structure are relatively conserved (19). The functional importance of the V3 region was demonstrated by a deficiency in the replication of V3-deletion viruses (20), and anti-V3 responses were early associated with fewer mother-to-child transmissions (21). The V2 region, which contains the a4b7 binding motif (22), forms a double loop with the V1 region and varies strongly in length, but contains some degree of sequence and structure conservation (19). While the V3 region in the HIV Env gp120 is strongly immunogenic and induces antibodies in essentially all HIV-infected individuals (10, 23), some of which can neutralize HIV-1 diverse strains, the V2 region only induces antibody responses in about 20–45% of infected individuals (10, 24).

A thorough understanding of vaccine-induced IgG recognition of antigenic regions and their variants within the HIV-1 Env might inform rational immunogen and vaccination schedule design. To this end, we here analyse the magnitude and variant breadth of the IgG response toward linear HIV-1 Env epitopes in HIVIS03/06 vaccinees. We have previously demonstrated that the multi-clade, multigene HIVIS-DNA priming, and heterologous recombinant modified vaccinia virus Ankara (MVA-CMDR) boosting regimen applied in the HIVIS03 trial elicited high frequencies of potent and durable antibody responses (25, 26). Neutralizing antibodies were not detected in the TZM-bl neutralization assay, however, in an infectious molecular clone (IMC)-PBMC assay, sera of up to 83% of vaccinees showed neutralizing activity (25, 26). ADCC-mediating antibodies were detected in the majority of vaccinees (97%) (26) and—in contrast to the waning antibody-responses in RV144 (16, 17)—were still present in 84% of vaccinees 3 years after the last vaccination (27). In the HIVIS06 trial, a late third MVA-CMDR boost, given after 3 years (between 2.7 and 3.2 years), successfully boosted HIV-1-specific humoral and cellular immune responses amongst the vaccinees (27). We here set out to dissect the antibody responses induced by the initial combined HIVIS-DNA/MVA-CMDR vaccination and the late third MVA-CMDR boost in more detail to elucidate whether the HIVIS03/06 vaccination schedule can induce and sustain antibody responses to HIV-1 Env epitopes associated with reduced infection risk in RV144 (9, 10).

## Materials and Methods

### Ethics Statement

The HIVIS03 and HIVIS06 trial protocols were approved by the Tanzania National Health Research Ethics Committee and the Senate Research and Publications Committee of the Muhimbili University of Health and Allied Sciences (MUHAS), as well as by the Regional Ethics Committee, Stockholm, Sweden. The use of the vaccine candidate products for humans was approved by the Tanzania Food and Drugs Authority. The trials were conducted in accordance with the International Conference on Harmonization Good Clinical Practice guideline. Written informed consent was obtained from all volunteers before enrolment.

### Study Design

**Figure 1:**
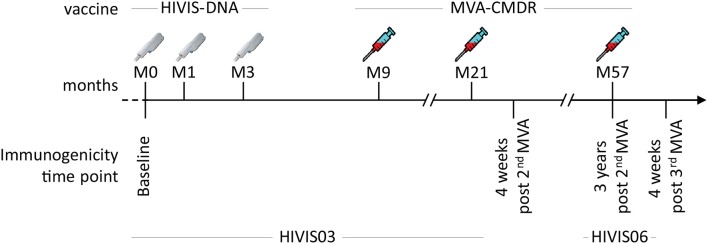
Summary of the HIVIS03/06 vaccination schedule. The HIVIS03/06 vaccination regimen included three injections of HIVIS-DNA (3.8 mg i.m./immunization or 1 mg id/immunization using Biojector), composed of 7 plasmids (encoding for gp160 Env subtypes A, B, and C, Rev subtype B, and Gag subtypes A, B, and RTmut). This was followed by two injections of 108 pfu i.m. of MVA-CMDR (CRF01_AE) coding for a membrane-anchored, functional HIV Env (subtype E), as well as Gag and Pol (subtype A) (25). An additional MVA-CMDR boost 3 years later concluded the vaccination schedule [HIVIS06 (27)].

In the HIVIS03 trial, a phase I/II clinical trial, conducted in Dar es Salaam, Tanzania among healthy adult volunteers, 60 HIV-uninfected volunteers were randomized into three groups of 20 volunteers to receive either placebo, 1 mg HIVIS-DNA intradermally (i.d.), or 3.8 mg intramuscularly (i.m.) prime. HIVIS-DNA plasmids expressing HIV-1 gp160 subtypes A, B, C; Rev B; Gag A, B, and RTmut B (28) were given at months 0, 1, and 3 using a needle-free Biojector device (25). This was boosted in the non-placebo groups by a recombinant MVA-CMDR encoding CRF01_AE derived Gag-Pol subtype A and a membrane-anchored functional HIV-1 gp150 Env subtype E (MVA-CMDR) that was administered at a dose of 10^8^ p.f.u i.m. by needle at months 9 and 21 (25) (Figure 1). The HIVIS06 trial was built upon the HIVIS03 trial, in which 20 volunteers, who had received 3 HIVIS-DNA and 2 MVA-CMDR immunisations in the HIVIS03 trial, were again recruited to receive an additional late 3rd MVA-CMDR vaccination, 3 years after the 2nd MVA-CMDR immunization (27). Ten of these 20 selected vaccinees had received 1 mg HIVIS-DNA i.d. and the remaining 10 had received 3.8 mg HIVIS-DNA i.m in the initial HIVIS03 trial. All samples were stored at −80°C until the time of testing. Safety and immunogenicity of the HIVIS03/06 vaccines were previously assessed in mice (28–31) and humans (25, 27, 32). In the present study, we used plasma samples collected from 20 vaccinees pre-vaccination (baseline), 4 weeks post 2nd MVA-CMDR vaccination, at the time of the 3rd MVA-CMDR vaccination, i.e., 3 years after the 2nd MVA-CMDR boost, and 4 weeks after the 3rd MVA-CMDR vaccination (Figure 1).

### Peptide Array Mapping of the HIV Env-Specific IgG Antibody Response

The peptide array design has been previously described in detail by our group (11). In brief, gp120 and gp41 sequences of 8 recently transmitted HIV primary isolates of different subtypes (A, B, C, CRF01_AE and CRF02_AG) were selected for inclusion in the peptide array design to represent the HIV Env variants of the current global pandemic. Additionally, two HIV Env vaccine sequences—CN54gp140 (subtype C) and CMDR (subtype AE)—were incorporated in the array. Previously identified hot spots of IgG recognition on the envelope (10, 11) were covered by up to 90 additional peptide variants [V2 (HxB2 163-177), V3 (HxB2 300-324), V4 (HxB2 409-447), gp41 immunodominant region (HxB2 576-614), and transmembrane cytoplasmic tail (HxB2 696-730)]. Each individual linear overlapping 15mer peptide on the array was present in triplicate.

**Figure 2:**
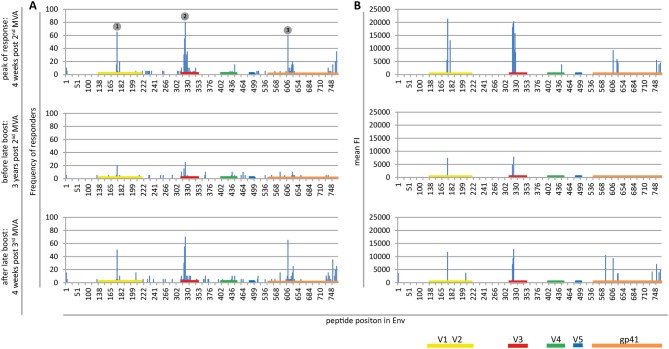
Maps of linear IgG epitopes along the HIV-1 Env targeted by HIVIS03/06 vaccinees with frequency of responders **(A)** and mean FI **(B)** given for each time point tested. FI values of each peptide were mapped to the 10 full-length Env sequences included in the array. The maximum FI at each peptide position is then used as a basis for the calculation of the frequency of responders. IgG responses against individual antigenic regions were considered positive if the corresponding maximum FI was above 2,500 after subtraction of the pre-vaccination value. The mean FI was calculated from all vaccinees for peptide position-specific IgG responses occurring in at least 10% of vaccinees. Numbered dots mark immunodominant Env regions, summarized in Table 1. FOR and mean FI at 4 weeks post 2nd MVA separated by subtype are depicted in Supplementary Figure 2.

**Figure 3:**
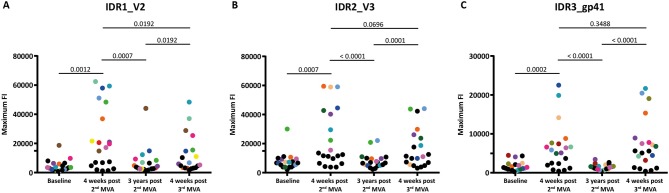
Boosting after 3 years with a 3rd MVA-CMDR significantly restores diminished immune responses to all 3 IDRs. The absolute maximum FI of each vaccinee to all variants (86 for HXB2 aa163, 36 for HXB2 aa304, and 21 for HXB2 aa580) corresponding to the respective HIV-1 Env position **(A)** IDR1_V2, **(B)** IDR2_V3, and **(C)** IDR3_gp41 is given without subtraction of the baseline response. Each dot represents one vaccinee. Statistical analysis of changes in the anti-Env IgG response against each IDR over all time points analyzed was conducted using the Wilcoxon matched-pair signed rank test. To showcase antibody-responses of selected individual vaccinees over time and IDRs, we highlighted vaccinees that exhibited high maximum FI 4 weeks post 2nd MVA-CMDR by assigning a unique color to them. All other vaccinees were assigned black dots.

Plasma from 20 HIVIS03/06 volunteers was analyzed using the peptide microarrays according to the manufacturer's instructions with minor modifications (www.jpt.com) as described elsewhere (11). Briefly, after initial blocking of the array slides, plasma samples were diluted 1:100 and incubated for 2 h at RT. Human IgG bound to the array was then detected using a secondary mouse anti-human-IgG Dylight649 antibody (1:5,000, 1 h at RT; JPT). Plasma from all visits of one vaccinee was processed simultaneously on the same day. After scanning the microarrays with a GenePix 4000A scanner at 650 (signal) and 532 nm (background) the resulting tiff files were analyzed using GenePix Pro 6.0 (Molecular Devices) by adding the array layout with an array-specific gal file. The layout was then controlled manually for accuracy. Results were exported from GenePix Pro 6.0 as gpr files, which link each position on the array with a fluorescence intensity (FI) value. These were processed using R scripts to first calculate the mean FI from the triplicate peptides and then to combine the information of each vaccinee at different time points. The resulting FI was then linked with the corresponding peptide sequences from a fasta file, containing the 10 full-length Env sequences included in the array. IgG responses against individual peptides were considered positive if the corresponding triplicate FI value was above 2,500 after subtraction of the pre-vaccination value (Figure 2). Mean FI values of all participants were calculated, if at least 10% of the vaccinees showed a positive response against the individual peptide. Immunodominant antigenic regions (IDRs) (Table 1) were defined as being recognized by at least 50% of volunteers at 4 weeks post 2nd MVA-CMDR. For statistical analysis (Figure 3), the maximum response of each vaccinee to all variants of the respective position without subtraction of the baseline response was used.

**Table 1:** Summary of immunodominant antigenic regions (IDR).

**IDR**	**Peptide position**	**HXB2 position**	**Env region**	**Representative sequence**	**FOR (%)**			**Mean FI**		
					**4 weeks post 2nd MVA**	**3 years post 2nd MVA**	**4 weeks post 3rd MVA**	**4 weeks post 2nd MVA**	**3 years post 2nd MVA**	**4 weeks post 3rd MVA**
IDR1_V2	176	164	V2	ELRDKKQKVHALFYK	65	20	50	21,257	7,323	11,614
IDR2_V3	325	304	V3	RKSIRIGPGSTFYAT	55	5	55	19,441	–	9,221
	326	305	V3	KSVRIGPGQTFYATG	80	25	70	20,287	7,765	12,669
IDR3_gp41	612	580	gp41	VLAVERYLKDQKFLG	60	0	65	9,248	–	9,247

*IDRs were defined as being recognized by at least 50% of volunteers at 4 weeks post 2nd MVA-CMDR. Identity of the immunodominant peak (see Figure 2), with corresponding peptide array and HXB2 Env amino acid starting position, and a representative amino acid sequence. For each time point investigated here, the FOR and mean FI is stated. FOR, frequency of responders; FI, fluorescence intensity*.

### Generation of phylogenetic Heat Maps and Sequence Logos of Env IgG Recognition

Maximum likelihood phylogenetic trees of the peptide variants corresponding to HxB2 amino acid positions 163 and 304 were generated using MEGA. The FI of each peptide variant included in the Env peptide array for the V3 and V2 tip (HxB2 163 and 305) has been linked with their phylogenetic relationship as described previously (11). The mean FI of all vaccinees for each peptide variant is color coded and the frequency of occurrence of a given peptide variant in the global HIV epidemic (www.hiv.lanl.gov) is depicted by its icon size. Phylogenetic heat maps were generated using R version 3.5.1.

Amino acid sequence logos depicting the amino acid probability pattern at given Env positions (Figure 5) were generated using WebLogo3 software (33).

### Statistical Analysis

Statistical analysis of the maximum FI against the V2 and V3 tip (Figure 3) was carried out using GraphPad Prism version 6. The Wilcoxon matched-pair signed rank test was used to compare the maximum fluorescence intensity between the different time points. A two-sided *p*-value of < 0.05 was considered statistically significant.

## Results

### The HIVIS Vaccination Regimen Induces IgG Responses Against the V2 and V3 tip as Well as Against gp41

Mapping of antigenic regions targeted by vaccine-induced Env-specific IgG responses was conducted in 20 participants of the HIVIS03 trial, receiving 3 HIVIS-DNA priming immunizations and 2 boosts with MVA-CMDR. The frequency and magnitude of the IgG response against individual linear overlapping peptides covering the HIV envelope after priming with HIVIS-DNA and boosting with MVA-CMDR are shown in Figures 2A,B upper row. Individual antibody responses of each vaccinee are depicted as a heat map in Supplementary Figure 1. Four weeks after the 2nd MVA-CMDR boosting, 3 IDRs within the V2, V3, and gp41 region of the HIV-1 Env, recognized by at least 50% of vaccinees, became apparent (Figure 2A upper row). Responses to all 3 IDRs increased significantly (*p* < 0.01) 4 weeks post 2nd MVA-CMDR as compared to baseline (pre-vaccination) (Figure 3). The IDRs, recognized by at least 50% of vaccinees and their corresponding HXB2 position, as well as the frequency of responders (FOR) and mean FI, are summarized in Table 1.

In the V2 region, the most frequently targeted peptide position (65% of participants; 13/20) corresponded to HXB2 aa164-178 (ELRDKKQKVHALFYK) (Table 1). An additional peptide within the V2 loop, corresponding to HXB2 aa168 (KKQKVHALFYKLDIV) and consisting of a highly conserved region including the α4β7 integrin-binding motif LDI/V (22), was recognized in 20% of the vaccinees (mean FI = 13,012). The IgG epitope targeted in the V3 region, was covered by two overlapping 15mer peptides corresponding to HXB2 aa304-319 and aa305-320, which were targeted in up to 55 and 80% of vaccinees, respectively. A further epitope located in the V3 loop, HXB2 aa311-324 was targeted in 35% of HIVIS03 recipients. IDR3_gp41, corresponding to HXB2 aa580-594 (VLAVERYLKDQKFLG), which partly covers the gp41 immunodominant region, was recognized in 60% of HIVIS03 vaccinees after the 2nd MVA-CMDR. Additionally, peptides corresponding to HXB2 aa727-741 in the gp41 cytoplasmic tail were targeted in 35% of vaccinees (Figure 2A upper row). Sixty percent of all vaccinees responded to both, IDR1_V2 and IDR2_V3 and 45% to all three IDRs. Only 1 vaccinee (5%) did not elicit an IgG response to any of the peptides in the array (Supplementary Table 1).

No significant difference in the vaccine-induced anti-HIV-1 Env IgG response between the 3.8 mg i.m. immunization and the 1 mg id immunization of the HIVIS-DNA could be observed (data not shown). Vaccinees of both injection groups showed the same pattern of Env recognition and antigenic regions were targeted to comparable levels.

### Boosting With a Late 3rd MVA-CMDR Restores Env-Specific IgG Responses Toward Identical Antigenic Regions as the Original HIVIS-DNA/MVA-CMDR Vaccination

In order to evaluate the durability of the HIV-1 Env-specific IgG response described above and the effect of a late boost with MVA-CMDR, we mapped HIV-1 Env antigenic regions in sera of the same 20 participants at 3 years after completing the HIVIS03 regimen and after the late boost with MVA-CMDR. Three years after the 2nd MVA-CMDR boosting of vaccinees in the HIVIS03 study, IgG response rates against linear HIV Env epitopes had declined considerably to only 20% against IDR1_V2, 5 and 25% against IDR2_V3, and 0% against IDR3_gp41 (Figure 2A and Supplementary Figure 1 middle row), with sera from 4 vaccinees (20%) completely failing to recognize any of the presented Env peptides (Supplementary Table 1). The magnitude of the response for all 3 IDRs also declined significantly (*p* < 0.01) (Figure 2B middle row and Figure 3). However, the late 3rd MVA-CMDR boosting (HIVIS06), restored the overall pattern of HIV-1 Env IgG recognition to an almost identical pattern as the one seen at 4 weeks after the 2nd MVA-CMDR immunization (HIVIS03), albeit at a lower magnitude (Figures 2A,B and Supplementary Figure 1 lower row). The FOR to the V2 loop was raised again to 50 and 70% to the V3 loop following the 3rd MVA-CMDR boost. The response against the gp41 immunodominant region, undetectable 3 years after the 2nd MVA-CMDR, was boosted by the late 3rd MVA-CMDR to a similar frequency (65%) and magnitude (9,247 mean FI) as after the 2nd MVA-CMDR. The increase in the magnitude of the response after the late 3rd MVA-CMDR was significant to all IDRs (*p* < 0.05) (Figure 3). We observed that vaccinees with a distinct IgG response against one of the IDRs after the 2nd MVA-CMDR, tended to respond against the same epitope after the late 3rd MVA-CMDR boost (colored dots in Figure 3). After the late boost, 50% of all vaccinees responded to IDR1_V2 and IDR2_V3 and 45% to all three IDRs. Only 2 vaccinees (10%) did not show a response to any of the Env peptides in the array (Supplementary Table 1). In summary, our data shows that the late 3rd MVA-CMDR boost restores linear anti-Env IgG responses to the same antigenic epitopes as the initial combined HIVIS-DNA/MVA-CMDR vaccination to near post 2nd MVA-CMDR levels.

### Comparable Antigen Variant IgG Recognition Patterns Are Detected After the Late 3rd MVA-CMDR Boost and the Original HIVIS-DNA/MVA-CMDR Vaccination

**Figure 4:**
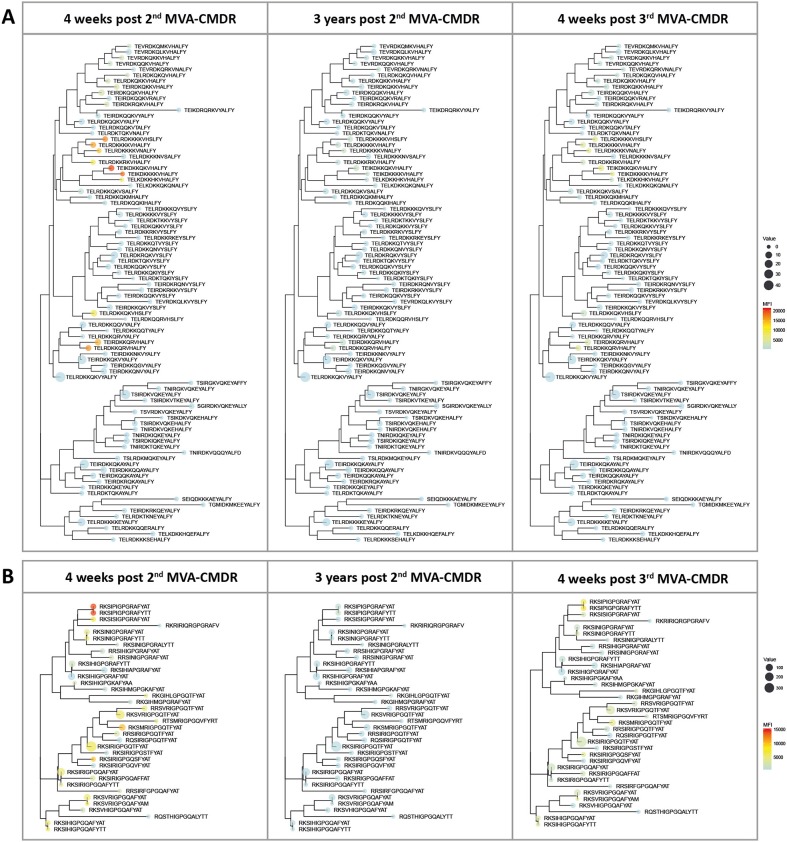
Phylogenetic heat map of IDR_V2 and IDR_V3 peptide variants targeted by vaccine-induced antibodies in HIVIS03/06 vaccinees. The strength of antibody-responses of all 20 vaccinees tested against each peptide variant (mean FI) corresponding with either IDR1_V2 or IDR2_V3, both of which were correlated to a reduced risk of HIV-infection in RV144, was calculated for each time point. The mean FI is illustrated in the context of their phylogenetic relationship, as well as their frequency of occurrence in the HIV database. The color of the dots represents the strength of the IgG response toward the given peptide, with a strong reaction displayed in red and a weaker reaction being displayed in blue. The icon size corresponds to the frequency of this peptide within the global HIV epidemic (www.hiv.lanl.gov) with a larger dot indicating a greater prevalence of the peptide. **(A)** Phylogenetic tree of 86 peptide variants corresponding to the HxB163_TGMIDKMKEEYALFY V2 position. **(B)** 36 peptide variants were tested for the V3 tip region (HxB304_RKSIRIGPGSTFYAT).

Inclusion of additional peptide variants at previously identified hot spots of IgG recognition of the HIV-1 Env in the peptide array design allowed fine mapping of the vaccination-induced IgG responses of the V2 and V3 tip (11), both correlated with a decreased risk of HIV-1 infection (10). This thereby enables a direct comparison of the variant recognition after the initial combined HIVIS-DNA/MVA-CMDR vaccination and the late 3rd MVA-CMDR boost. The V2 loop (HXB aa163_ TEIKDKKQKVHALFY, Figure 3A) was covered by 86 peptide variants and the V3 tip (HXB aa304_ RKSIRIGPGSTFYAT, Figure 3B) by 38 peptide variants. The mean FI of all 20 vaccinees per time point (4 weeks post 2nd MVA-CMDR, 3 years post 2nd MVA-CMDR, and 4 weeks post 3rd MVA-CMDR) was calculated for each peptide variant included in the array and projected as a heat map onto a phylogenetic tree illustrating the relationship of the peptide variants as well as their frequency within the global HIV epidemic (Figure 4). HIVIS03/06 volunteers produced Env-specific IgG responses toward several different peptide variants of the V2 tip (HxB2 aa163), with recognition of two clusters of closely related variants TE(/I)LRDKKK(/R/Q/H)KVHS(/A/N/H)LFY and TEI(/L)RDKKQRVHALFY, with one outlier (TELRDKKQKVHSLFY) (Figure 4A). Variant TEIKDKKQKVHALFY was the most strongly recognized at all time points (Figure 4A). The corresponding, but non-analogous, MVA-CMDR vaccine sequence ELRDKKQKVHALFYK, present on the array at HxB2 position 164 due to differential cleavage, was similarly strongly recognized at 4 weeks post 2nd MVA-CMDR (mean FI: 13,746), but not as strongly boosted after the 3rd MVA-CMDR (mean FI: 3,830) (data not shown). For the V3 tip (HXB aa304), we observed a broader response with recognition of several different variants with less clustering of positive responses among closely related sequences (Figure 4B). This response decreased 3 years after the 2nd MVA-CMDR but was re-established to some extent following the single dose of the late 3rd MVA-CMDR (Figure 4B). The peptide variant most strongly recognized at all 3 time points tested was RKSIPIGPGRAFYTT. The corresponding MVA-CMDR sequence (HxB2 aa307: TSIPIGPGQAFYRTG) was recognized equally well as the V3 variant RKSIPIGPGRAFYTT at all 3 time points (mean FI 4 weeks post 2nd MVA-CMDR: 14,825, mean FI 3 weeks post 2nd MVA-CMDR: 702, mean FI 4 weeks post 3rd MVA-CMDR: 7,460) (data not shown).

Of note, the V2 and the V3 loop sequence variants most strongly recognized here were not the most frequent in the global HIV epidemic as defined by occurrence in the HIV Los Alamos database (www.hiv.lanl.gov) and depicted in Figure 4 by icon size.

For the relatively conserved IDR3_gp41, with only 21 peptide variants present on the array, only one sequence variant (VLAVERYLKDQKFLG) was recognized at both time points (data not shown).

These data show that a single late MVA-CMDR boost can restore IgG-responses toward the same peptide variants as those that were targeted by the combined initial HIVIS-DNA/MVA-CMDR vaccination, even if administrated after a 3-year interval.

### IDR1_V2 and IDR3_gp41 Responses Are Restricted to Variants With Close Homology to the MVA-CMDR Immunogen Sequence, While IDR2_V3 Responses Are More Cross-Reactive

**Figure 5:**
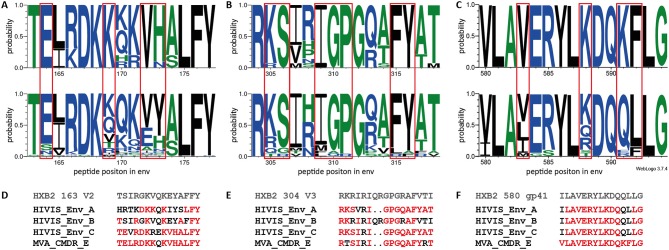
Amino acid probability analysis for the three IDRs shows preferential targeting of certain amino acids by vaccine-induced IgG. Sequence logos depicting the amino acid probability pattern of the most strongly recognized peptides (mean FI >5,000) at 4 weeks after 2nd MVA-CMDR for HXB2 163 V2 (*n* = 10) (**A** upper row), HXB2 304 V3 (*n* = 14) (**B** upper row), and HXB2 580 gp41 (*n* = 1) (**C** upper row) IDRs. The lower row represents peptide variants with a mean FI below threshold (<2,500) for IDR1_V2 (**A**; *n* = 70), IDR2_V3 (**B**; *n* = 14), and IDR3_gp41 (**C**; *n* = 20). The height of the letter indicates the probability of an amino acid occurring at a given position. Red boxes highlight amino acids with preferred targeting after vaccination. Amino acids are colored according to their hydrophobicity (hydrophilic—blue; neutral—green; hydrophobic—black). **(D–F)** Vaccine and HXB2 sequences corresponding to the 3 IDRs. Amino acids with a ≥0.6 probability of targeting in the HIVIS03 vaccine regimen are highlighted in red.

To determine the effect of the different immunogens on the elicited antibody response, we analyzed preferred targeting of certain amino acid motifs of the 3 IDRs in the context of the immunogen sequences (Figure 5). A direct comparison of strong to moderately recognized (mean FI >5,000; *n* = 10) and non-recognized (mean FI <2,500; *n* = 70) peptide variants in the V2 loop (HXB2 aa163), at 4 weeks after the 2nd MVA-CMDR, corresponding to IDR1_V2, showed a distinct preference of E^164^, K^169^, and VH^172−173^ of the HIVIS vaccine-induced IgG response (Figure 5A). The amino acids with a probability of recognition of >0.6 closely match the MVA-CMDR immunogen sequence (Figures 5A,D). Dissecting the antibody-targeting of the HIV-1 Env by HIV-1 subtype and vaccine also shows a strong preference of the MVA-CMDR sequence at IDR1_V2, followed by sequences representative for subtype AG and C (Supplementary Figure 2). Representative IDR1_V2 peptides sequences, which had a strong recognition (mean FI >2,500) where aligned against the HIV Los Alamos database. Only a small number of these sequences showed a close homology to our peptides (Supplementary Figure 3). The reactive peptides recognize mainly subtype AE and C sequences, reflecting the subtype of the MVA-CMDR, however, there is no difference in the homology profile of highly reactive and non-reactive sequence pairs. The IgG-response toward the V3 was more cross-reactive than the V2 response with a total recognition of 22 out of 36 peptide variants. Comparison of strong to moderately recognized (man FI >5,000; *n* = 14) variants to non-recognized variants (mean FI <2,500; *n* = 14) of the V3 loop (represented by HXB2 aa304) revealed a preferred recognition of amino acids KS^305−306^, IGP^309−311^, and FY^315−316^ (Figure 5B). Amino acids targeted with a high probability (>0.6) match relatively close to the MVA-CMDR as well as two out of the three HIVIS-DNA plasmids (subtypes A and C, but not B) immunogen sequences (Figures 5B,E). This broad recognition of V3 epitopes of various subtypes is shown in Supplementary Figure 2, where high percentages of vaccinees elicit IgG responses against sequences representing subtype C, followed by MVA-CMDR, subtype AG, and then subtypes B and A. Similar results can be seen from the homology profile of representative IDR2_V3 peptides in Supplementary Figure 3, where each peptide shows homology to a large number of sequences and all subtypes are represented. Even within the relatively conserved IDR3_gp41 (HXB2 aa580), partly covering the gp41 immunodominant region, a vaccine-induced preference of IgG targeting peptide variants with V^583^, K^588^, and KF^591−592^ could be observed (Figure 5C). Here, only one sequence variant (VLAVERYLKDQKFLG), out of 21 included in the array was recognized 4 weeks after the 2nd MVA-CMDR (mean FI: 5,554) and the 3rd MVA-CMDR (mean FI: 6,455). The recognized sequence was an exact match to that of the MVA-CMDR subtype AE immunogen sequence (Figure 5F and Supplementary Figure 2). As described in paragraph 3.3, peptide variant recognition after the 3rd MVA-CMDR closely matched recognition after the 2nd MVA-CMDR.

The comparison of peptide variants preferentially targeted by HIVIS03/06 vaccinees with the corresponding immunogen sequences revealed a strong influence of the MVA-CMDR vaccine for the IgG recognition of IDR1_V2 as well as IDR3_gp41, where the amino acid sequences of preferred peptides closely match the MVA-CMDR immunogen sequence. IgG targeting of IDR2_V3, on the other hand, was more cross-reactive and less constrained to one of the immunogen sequences.

## Discussion

In the present study, we assessed the magnitude and cross-reactivity of the IgG antibody response against linear HIV-1 Env epitopes induced by a heterologous multi-clade, multigene HIVIS-DNA prime and heterologous MVA-CMDR boost vaccine regimen (25), using a linear peptide array spanning the complete HIV-1 Env. We further analyzed the effect of a late boosting injection with solely MVA-CMDR (27) on restoring the anti-HIV-1 Env IgG response to comparable magnitudes and antigenic variant recognition.

We demonstrate that the HIVIS03 vaccination regimen induced IgG responses against linear epitopes within the V2 and V3 tip, both associated with a reduced risk of HIV infection in the RV144 trial (9, 10), as well as the gp41 immunodominant region. Antibody responses against the V2 loop and the gp41 immunodominant region were relatively narrow and more pronounced against peptide variants closely resembling the MVA-CMDR immunogen sequence rather than the HIVIS-DNA sequences used for priming, whereas the anti-V3 response was more cross-reactive. Three years after the second MVA-CMDR boost, these HIV Env-specific antibody responses had declined significantly, however boosting with a late third MVA-CMDR in HIVIS06 restored IgG responses to the same linear Env epitopes and antigenic variants. This finding has potential implications for HIV-vaccine design, as it shows that a single boost with MVA-CMDR can sustain anti-Env antibody responses, associated with a reduced risk of infection in RV144 (9, 10) as well as in an SIV challenge NHP model (14).

The HIV-1 Env linear B cell epitopes (IDR1_V2, IDR2_V3, and IDR3_gp41) detected here in HIVIS03/06 participants are similar to those recognized in TaMoVac I vaccinees after HIV-DNA priming and MVA-CMDR boosting using the same peptide microarray (11). The TaMoVac I vaccinees received the same HIV-DNA and MVA-CMDR immunogens used here, with i.d. HIV-DNA immunizations delivered at weeks 0, 4, 12, and 10^8^ pfu HIV-MVA given i.m. at weeks 30 and 46. TaMoVac I was designed to evaluate a simplified DNA vaccination regimen and compared 5 injections of HIV-DNA, 1,000 μg total dose (3 Env and 2 Gag encoding plasmids) with two “simplified” regimens of 2 injections of HIV-DNA, 600 μg total dose, of Env- and Gag-encoding plasmid (34). Additionally, the TaMoVac I vaccinees received two boosts of CN54rgp140/GLA-AF protein 4 weeks apart 30–71 weeks after the last MVA-CMDR vaccination (35). Both vaccine trials induced IgG antibody responses toward linear epitopes located in the V2 and V3 loop as well as in the gp41 immunodominant region. Boosting with CN54gp140 protein in TaMoVac I recipients resulted in a higher magnitude and breadth of the V3 response, as well as in the recognition of additional Env regions, which are, however, mostly inaccessible on a native trimer (11). Interestingly, the V2 response in TaMoVac I vaccinees was not affected by the protein boost and was also focussed on peptide variants with close homology to the MVA-CMDR immunogen sequence. Antibody responses toward the same area in the V2 loop—N-terminal to the α4β7 binding motif—as detected in HIVIS03/06 and in TaMoVac I vaccinees, were also detected in RV144, RV305, VAX003, and HVTN100 vaccine recipients, but not in VAX004 and UKHVC 003SG vaccinees, and only in few HIV-1 infected subjects (10, 11, 23, 36–39). In the RV144 trial, this V2-specific IgG response was associated with a reduced risk of HIV infection (10), however, no reduced risk of infection was seen in the VAX003 trial, which might have been due to differences in IgG subclasses of the antibodies specific for the V2 loop crown (16, 40). While the anti-V2 response in RV144 was dominated by IgG3 antibodies, IgG4 antibodies prevailed in VAX003.

Interestingly, the late boost consisting of a single dose of MVA-CMDR employed here in the HIVIS06 vaccination schedule, not only induced recognition of the same antigenic epitopes but also the same peptide variants as detected following the original HIVIS-DNA/MVA-CMDR vaccination. We, therefore, compared preferably targeted peptide variants to the immunogen sequences used. This revealed that for both, IDR1_V2 and IDR3_gp41, only variants with close homology to the MVA-CMDR immunogen sequence were recognized. The IgG anti-V3 response, however, was much broader at both time points—recognizing several HIV-1 subtypes, which might be due to the fact that the V3 region is the least variable of the HIV-1 variable regions and therefore might be structurally more conserved (19).

Single amino acids can be critical for epitope formation and therefore antibody binding, as was reported in RV144 vaccinees, where K^169^ and V^172^ were critical for V2 loop binding by IgG (36, 37). The importance of K^169^ for IgG antibody binding was further demonstrated by its sieve effect on break-through viruses in RV144 (12). Interestingly, when applying the HIVIS03/06 vaccine regimen, where the MVA-CMDR immunogen V2 sequence is identical to the RV144 immunogens ALVAC-HIV and AIDSVAX E, also only peptide variants with K^169^ and V^172^ were targeted. All amino acid positions that proved to be crucial for targeting by antibodies elicited by the HIVIS03/06 vaccination regimen (E^164^, K^169^, and VH^172−173^) are located at Env positions with lower sequence conservation (13), which might explain the limited breadth of the V2 response detected here. This lower sequence conservation can also be observed in the homology profile representative IDR1_V2 sequences. Even though, the breadth of V2 response observed in HIVIS vaccinees seems narrower than Gottardo et al. reported for RV144 and VAX003 vaccinees, still a similar preference of peptides present in Env sequences of subtype AE (corresponding to the MVA-CMDR), AG, and subtype C can be observed. This leads to the conclusion that the immunogen sequence strongly influences IgG responses elicited by the immunogen and calls for optimal immunogen design to achieve broader anti-V2 responses. Amino acids that were important for V3 targeting in HIVIS03/06 vaccinees—especially IGP^309−311^ and FY^315−316^–on the other hand, are much more conserved (13), and thus might lead to a much broader IgG response, targeting various HIV-1 subtypes. In contrast to IgG responses targeting the V2 region, antibodies toward the V3 loop are present in essentially all HIV infected patients and human and animal model vaccine studies using immunogens that include the Env V3 region (10, 11, 24, 41–43). Presence of such anti-V3 antibodies in vaccinees with low levels of anti-Env specific IgA was also associated with protection in the RV144 trial (10).

Glycosylation patterns and conformational aspects of the immunogens will influence the accessibility of B cell epitopes and therefore direct the vaccine-induced antibody response. None of the 3 IDRs detected in HIVIS03/06 vaccinees contains glycosylation motifs and are thus more likely to be accessible. Furthermore, only antibodies targeting epitopes accessible on the native HIV-1 Env trimer will be able to bind in the natural course of infection and prevent infection. Mapping of the IDRs onto a 3D structure of a native-like Env trimer described in Nadai et al. (11), allowed us to infer the conformational location of the 3 IDRs detected here. Both IDR1_V2 and IDR2_V3 map to the trimer apex and are located on the surface of the trimer, while IDR3_gp41 would be hidden in the inter-protomer region of a native trimer. Yet, in the native-like membrane-bound, functional MVA-CMDR encoded gp150 immunogen, IDR3_gp41 lies close to the C-terminus, and might, therefore, be accessible.

An earlier study on the durability of immune responses induced by HIVIS03/06 vaccination (27) showed that 3 years after the 2nd MVA-CMDR 90 and 85% of the participants still had detectable ELISA binding antibodies to subtype C gp140 and subtype B gp160 antigen, respectively, albeit at significantly lower titres than at peak immunogenicity. In the present study, we show comparable 3-year durability of IgG antibodies targeting linear HIV-1 Env peptides, with 80% of vaccinees still recognizing any of the linear HIV-1 Env peptides presented by the microarray. When dissecting this total anti-Env IgG response into individual specificities, however, a strong variance in the durability of antibodies targeting discriminative epitopes can be observed. IgG antibodies to all three immunodominant linear HIV-1 Env epitopes elicited by the initial HIVIS03 vaccination show a significant decline 3 years after the second MVA-CMDR. Yet, when comparing classical protein-based ELISAs and linear peptide microarrays, advantages and limitations of each assay have to be considered. As only linear epitopes will be displayed on the peptide array, antibodies to conformational epitopes such as discontinuous (i.e., CD4-binding site) or quaternary epitopes (i.e., arising from Env trimerisation) will not be detected. Such discontinuous and structural epitopes might be present on the antigens used in ELISA assays and therefore, could lead to a higher sensitivity of the ELISA assays. Linear peptide microarrays in contrast to classical protein-based ELISAs, however, allow for the simultaneous analysis of the magnitude as well as the breadth of the IgG response toward multiple linear epitopes and is therefore suitable for high-throughput fine mapping of antibody specificities.

In the light of the parallel decline of vaccine efficacy (15) and anti-HIV-1 Env antibodies (16, 17) in RV144, the restoration of antibody responses to the V2 and V3 epitopes, associated with a reduced risk of infection, by repeated boosts would be desirable. Considering these findings, the sustainability of antibody responses to the V2 and V3 HIV-1 Env epitopes by a single dose of the MVA-CMDR vector immunogen instead of protein-based immunogens described in the present study might, therefore, have implications to the advancement of HIV-vaccine design. Regular protein boosts in the non-protective HIV vaccine trial VAX003 were shown to increase levels of total IgG anti-V2 antibodies, yet, did not improve magnitude or durability of V2 responses and led to a decline in anti-V2 IgG3 antibodies. A IgG3 dominated V2 response was associated with a reduced risk of HIV infection in RV144 (16, 44). Boosting of HIV-1 uninfected RV144 participants 6–8 years after the completion of RV144 in the RV305 trial showed promising results as an increase in the breadth of antibody effector functions in V2-specific antibodies as well as long durability of V2-specific memory B-cell clones could be detected (45). Yet, an analysis into anti-Env and anti-V1V2 antibody titres by vaccination group showed significant differences in the immunogens used (38). While immunisations solely with the ALVAC-HIV canarypox vector only slightly increased anti-gp70 V1V2 titres, they did not increase IgA responses to the HIV-1 Env (38), which previously were inversely correlated with infection risk in RV144 (10). Immunisations with the bivalent HIV-1 gp120 AIDSVAX B/E protein alone or in combination with ALVAC-HIV, on the other hand, led to significantly increased anti-gp70 V1V2 IgG levels, but, similarly to the VAX003 and VAX004 trials (16), simultaneously increased IgA responses to the HIV-1 Env (38). Potential IgG subclass changes induced by the MVA-CMDR boost are of interest. Studies of V1V2-specific IgG and IgG subclass responses in HIVIS03/06 vaccinees are reported separately (Joachim et al.; submitted).

In summary, combined heterologous prime-boost vaccination of HIVIS-DNA and MVA-CMDR induced strong anti-V2, V3 and gp41 immunodominant region IgG responses that were efficiently boosted—and targeted the same peptide variants—by a single injection of MVA-CMDR 3 years after the original vaccination. This indicates that antibody responses against the HIV-1 Env, potentially reducing the HIV-1 infection risk that were induced by the initial prime-boost schedule, can be boosted and maintained by repeated injections with a single dose of MVA-CMDR.

## Data Availability Statement

The datasets generated for this study are available on request to the corresponding author.

## Ethics Statement

The studies involving human participants were reviewed and approved by Tanzania National Health Research Ethics Committee and the Senate Research and Publications Committee of the Muhimbili University of Health and Allied Sciences (MUHAS), as well as by the Regional Ethics Committee, Stockholm, Sweden. The patients/participants provided their written informed consent to participate in this study.

## Author Contributions

AJ performed laboratory work and contributed to data analysis and interpretation as well as manuscript writing. MA performed laboratory work and contributed to data analysis. GP contributed to peptide array design and data analysis. LR contributed to data analysis and manuscript writing. VH programmed R scripts for phylogenetic heat maps. PM, SA, EL, MB, MR, and BW contributed to the clinical trials studies. ES, CN, and GB contributed to clinical trials study coordination and manuscript writing. CG conceived the study, contributed to data analysis and interpretation, as well as to manuscript writing. KH conceived the study, contributed to data analysis and interpretation, and wrote the manuscript. All authors reviewed and edited the manuscript.

## Conflict of Interest

The authors declare that the research was conducted in the absence of any commercial or financial relationships that could be construed as a potential conflict of interest.
