# Supplementary material for: Induction of Identical IgG HIV-1 Envelope Epitope Recognition Patterns After Initial HIVIS-DNA/MVA-CMDR Immunization and a Late MVA-CMDR Boost
Source: Front Immunol. 2020 Apr 28;11:719. doi: 10.3389/fimmu.2020.00719 (PMC7198863; doi:10.3389/fimmu.2020.00719)
Supplement: Supplementary file 1 [file Data_Sheet_1.pdf]

Supplementary Material

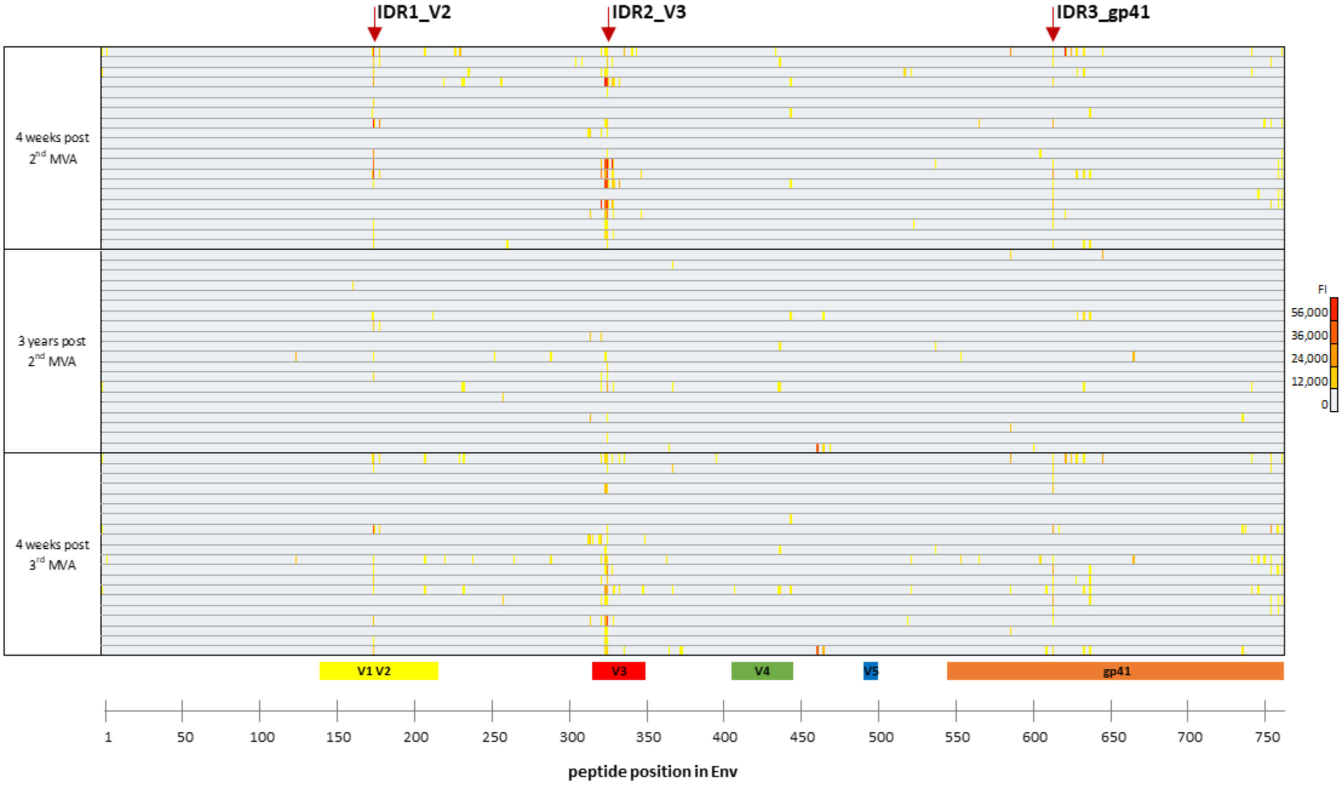

Supplementary Figure 1. Heat map of IgG responses against individual antigenic regions along the HIV-1 Env.

Maps of antigenic regions targeted by Env-specific IgG responses in each vaccinee at all three time points tested. Each row represents one vaccinee (n = 20 per time point). FI values corresponding to each peptide position were mapped to the 10 full-length Env sequences included in the peptide array (HIV primary isolates of different subtypes (A, B, C, CRF01_AE and CRF02_AG) and HIV Env vaccine sequences (CN54gp140 and CMDR)). Responses above 2500 FI after baseline subtraction were considered positive and the maximum FI was selected per position. The FI is shown for positive responses (>2500) for each vaccinee. The red arrows indicate the 3 IDRs.

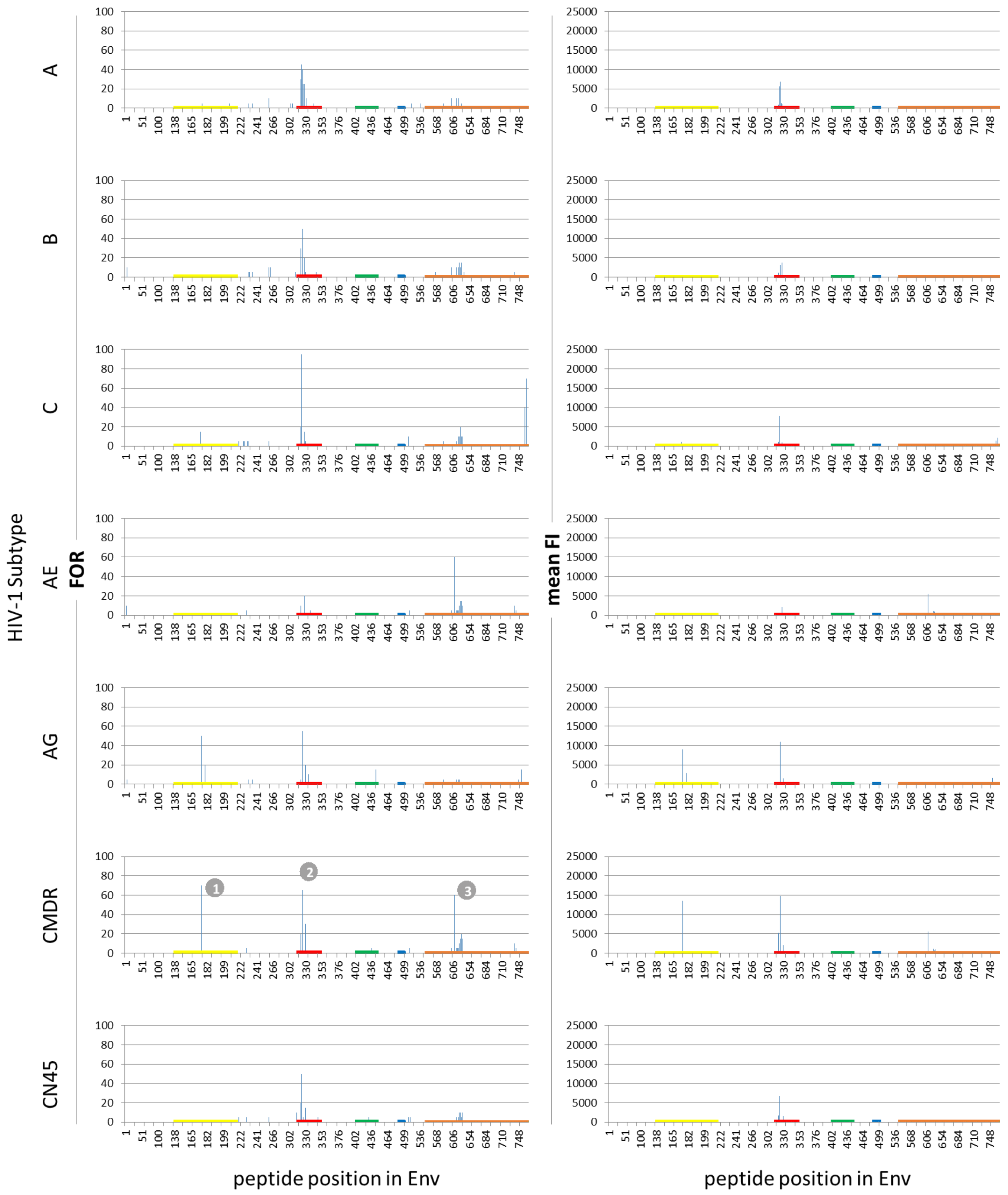

Supplementary Figure 2. Maps of linear IgG epitopes along the HIV-1 Env targeted by HIVIS03/06 vaccinees with a frequency of responders and mean FI at 4 weeks post 2nd MVA.

FI values of each peptide were mapped to the 10 full-length Env sequences included in the microarray separately for each subtype and vaccine sequence. IgG responses against individual antigenic regions

were considered positive if the corresponding FI was above 2500 after subtraction of the pre-vaccination value. The mean FI was calculated from all vaccinees for peptide position-specific IgG responses occurring in at least 10% of vaccinees. Bullet points in the graph illustrating CMDR-specific responses indicate the 3 IDRs. The representative subtype sequences of 8 recently transmitted HIV primary isolates included in the microarray (A: A1_AF407160__Q842-d12_PNS70d_KE, A1_GU481385__06_RU_SP_R163_IorII_13_RU; B: B_AF041133_PHI374_FR, B_US_1990_BORId9_2F8_EU576282; AE: 01_AE_FR_1996_PHI426_AY231158; AG: AG_AY231152_PHI127_FR; C: C_EF117273__HIV_25925_2_INl, C_MW_2003_CHV0011210_0393_C3_FJ444215) and two HIV Env vaccine sequences CN54gp140 (subtype C) and CMDR (subtype AE) were used for mapping.

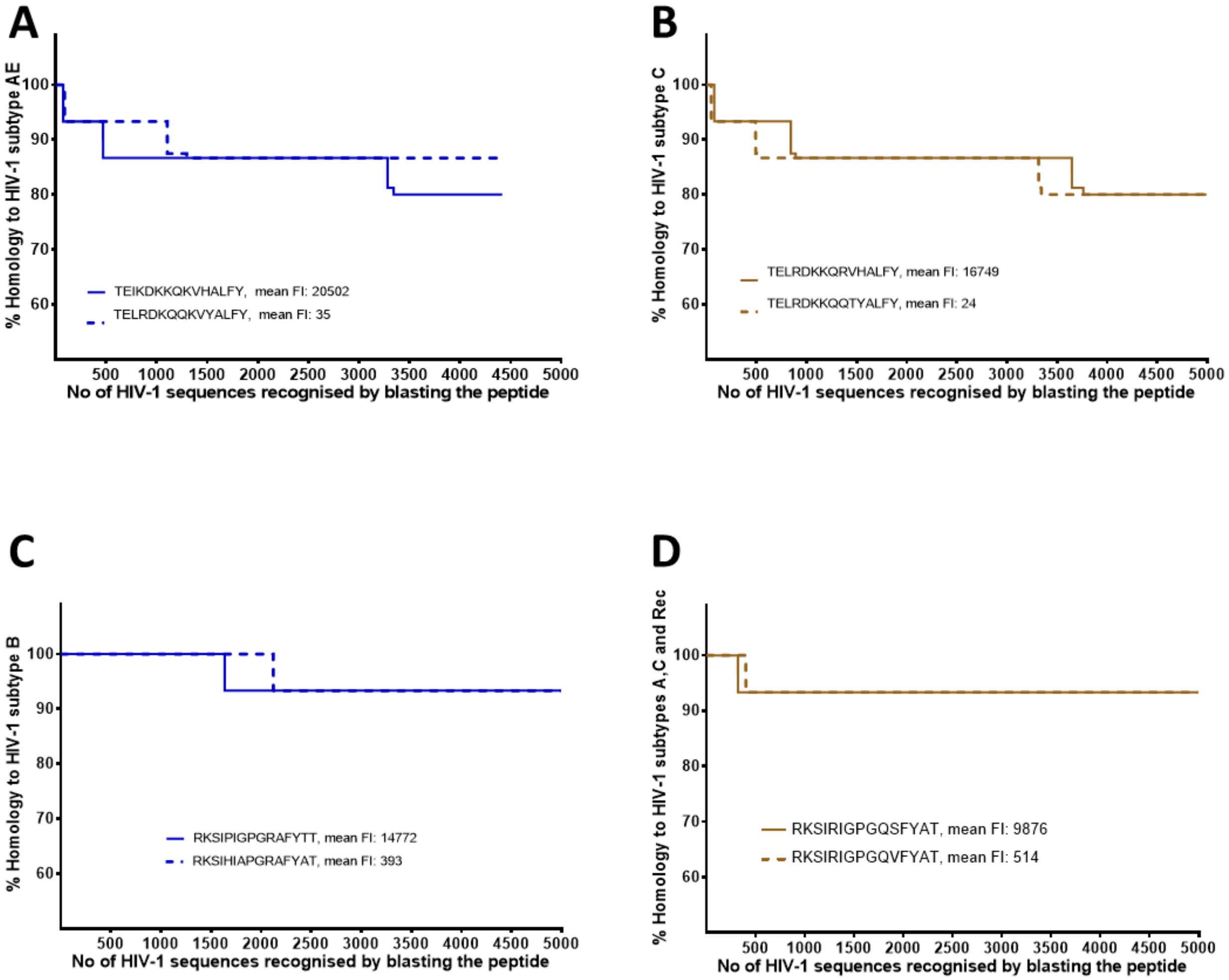

Supplementary Figure 3. Number of HIV-1 sequences with close homology to peptide variants with high and low recognition by HIVIS vaccinees for IDR1_V2 and IDR2_V3 by HIV-1 subtype.

Representative sequences of peptide variants with high (>2500) and low mean FI (<2500) across all 20 vaccinees were blasted (NCBI and HIV Los Alamos database (www.hiv.lanl.gov)) and the number of resulting HIV-1 sequences is blotted against the percent homology to the peptide variant. Graphs are shown for the subtypes with the highest homology for both IDRs. **A/B**: Representative sequences of IDR1_V2. **A**: Peptide sequences TEIKDKKQKVHALFY, mean FI: 20502 (solid line), TELRDKQQKVYALFY, mean FI: 35 (dotted line) with close homology to HIV-1 subtype AE. **B**: Peptide sequences TELRDKKQRVHALFY, mean FI: 16749 (solid line) and TELRDKKQQTYALFY, mean FI: 24 (dotted line) with close homology to HIV-1 subtype C. **C/D**: Representative sequences of IDR2_V3. **C**: Peptide sequences RKSIPIGPGRAFYTT, mean FI: 14772 (solid line) and RKSIHIAPGRAFYAT, mean FI: 393 (dotted line) with close homology to HIV-1 subtype B. **D**: Peptide sequences RKSIRIGPGQSFYAT, mean FI: 9876 (solid line), RKSIRIGPGQVFYAT, mean FI: 514 (dotted line) with close homology to HIV-1 subtypes A, C and their Recombinant forms.

Supplementary Table 1:

Percentage of vaccinees responding to one or multiple immunodominant antigenic regions (IDR) detected in the peptide microarray and HIV-1 Env-specific binding antibodies as measured by ELISA*. IDRs were defined as being recognised by at least 50% of volunteers at four weeks post 2nd MVA-CMDR. The FOR is shown in % vaccinees responding to each of the 3 IDRs as well as for multiple IDRs or any other Env regions. # IgG response to any of the two peptides was detected (HXB2 aa 304 and 305). For each time point investigated here, the FOR is stated. *Data on binding antibodies to HIV-1 IIIB subtype B gp160 or recombinant HIV-196ZM651 subtype C gp140 were taken from Joachim, et al. 2017 (1). FOR = frequency of responders.

	Peptide Microarray [FOR]							ELISA [FOR / median titre]*	
time point	IDR_V2	IDR_V3 1&2#	IDR_gp41	IDR_V2 & V3	IDR_V2, V3, & gp41	any other Env region	any Env region	HIV-1 subtype C gp140	HIV-1 subtype B gp160
4 weeks post 2nd MVA	65	80	60	60	45	85	95	100 / 3200	89 / 800
3 years post 2nd MVA	20	30	0	10	0	75	80	90 / 200	85 / 100
4 weeks post 3rd MVA	50	75	65	50	45	75	90	100 / 800	85 / 400

Supplementary References:

1. Joachim A, Munseri PJ, Nilsson C, Bakari M, Aboud S, Lyamuya EF, et al. Three-Year Durability of Immune Responses Induced by HIV-DNA and HIV-Modified Vaccinia Virus Ankara and Effect of a Late HIV-Modified Vaccinia Virus Ankara Boost in Tanzanian Volunteers. AIDS research and human retroviruses. 2017;33(8):880-8.
